# Absent Internal Carotid Artery With Intact Circle of Willis

**DOI:** 10.7759/cureus.17090

**Published:** 2021-08-11

**Authors:** James W Sonne, Helen Kaiser, Shanna Williams

**Affiliations:** 1 Biomedical Sciences, University of South Carolina School of Medicine Greenville, Greenville, USA

**Keywords:** agenesis, internal carotid artery, circle of willis, cerebral blood flow, hypophysis

## Abstract

The internal carotid arteries are one of the primary suppliers of the Circle of Willis and cerebral blood flow, but the rare case of agenesis of the internal carotid artery can impair the functional redundancies of cerebral blood supply.

In this study, routine, medical education-focused cadaveric dissection of an 80-year-old male cadaver (cause of death was ventricular tachycardia) was performed.

A case of agenesis of the left internal carotid artery and the carotid canal was identified. Upon investigation, we found that the compensatory pattern of irrigation in the Circle of Willis did not conform to previously described cases in the scientific literature. Further literature review suggested that such agenesis can be associated with a wide range of conditions from stroke, migraine, tinnitus, and Horner’s syndrome.

Due to the altered blood flow pattern, we caution the reading physician regarding the potential for ischemia and iatrogenic damage, particularly of the pituitary gland and eye. We suggest the use of neuroangiographic imaging in cases of agenesis of an internal carotid artery.

## Introduction

The internal carotid arteries bifurcate from the common carotid arteries to traverse the carotid canals and eventually provide blood supply, along with the vertebral arteries, to the brain by way of the Circle of Willis [1]. Branches directly from the internal carotid arteries supply a number of critical structures including the pituitary gland from the superior hypophyseal artery, and the orbit and eye from the ophthalmic artery. Collateral circulation from the bilateral internal carotid arteries and vertebral arteries provides redundant irrigation to the brain in cases of stenosis or temporary, acute occlusion during regular head movements.

Developmentally, the internal carotid arteries arise from the third aortic arch within the third pharyngeal arch. This third pharyngeal arch is also responsible for the development of a portion of the hyoid bone, the stylopharyngeus muscle, and the glossopharyngeal nerve (CN IX) responsible for motor and sensory innervation of portions of the visceral neck. The third aortic arch combines with the dorsal aorta of the aortic sac of the developing heart to eventually form the ascending, distal portion of the internal carotid artery [2]. As with any developmental event, functional or morphological errors can result. In the case of the internal carotid artery, hypoplasia or even complete agenesis has been known to occur [3].

Because of the numerous and sometimes tortuous branches of the cervical neck and internal carotid artery, the absence of the internal carotid artery can lead to compensation through hyperplasias, rare anastomoses, and fistulas [4]; while also being correlated with issues of cerebral circulation including aneurysm or thromboembolism [5]. In this article, we describe a case of left-sided agenesis of the internal carotid artery verified by absent ipsilateral carotid canal identified during routine cadaveric dissection for medical education. Hyperplasia of the vertebral, basilar, and contralateral internal carotid arteries was present; but no other morphological variations or remnant arteries were identified, and a complete Circle of Willis was present. While previous cases have been reported in the scientific literature, these features represent a unique presentation that does not appear to be previously described [4, 6-7]. Furthermore, we discuss potential anastomotic pathways to the orbit, eye, and hypophysis, along with the clinical relevance of these circulatory patterns.

## Case presentation

Routine dissection of 10 human cadavers was performed in the educational gross anatomy laboratory of the authors’ institution between August 2020 and January 2021. Rotations of M1 medical students, assisted by M4s and the anatomy module faculty performed the dissections guided by provided laboratory images. During the final unit of the module, head and neck dissection was performed following thoracic dissection and prior to craniotomy and brain removal.

**Figure 1:**
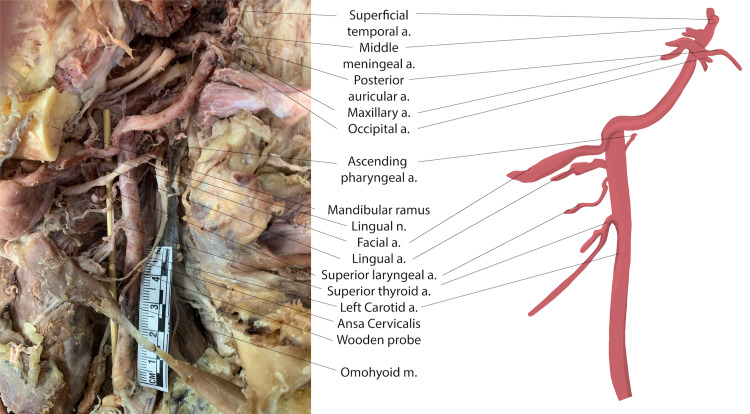
Dissection of the left neck. Dissection of the left neck revealing the complete agenesis of the left internal carotid artery. (Line art courtesy of Sara DeHaan.)

During routine dissection, a notable absence of the left internal carotid artery was identified (Figure 1) in an 80-year-old, male cadaver with ventricular tachycardia listed as the cause of death. The left common carotid artery branched from the aortic arch to ascend within the carotid sheath, eventually giving off all notable branches of the external carotid artery without exception. However, no bifurcation to form the left internal carotid artery was present. The gross dilation typically attributed to the carotid sinus was present at the level of the superior thyroid artery. Components of the third pharyngeal arch, including the stylopharyngeus and glossopharyngeal nerves, were identified bilaterally from a retropharyngeal view. The hyoid bone was present without notable variation.

**Figure 2:**
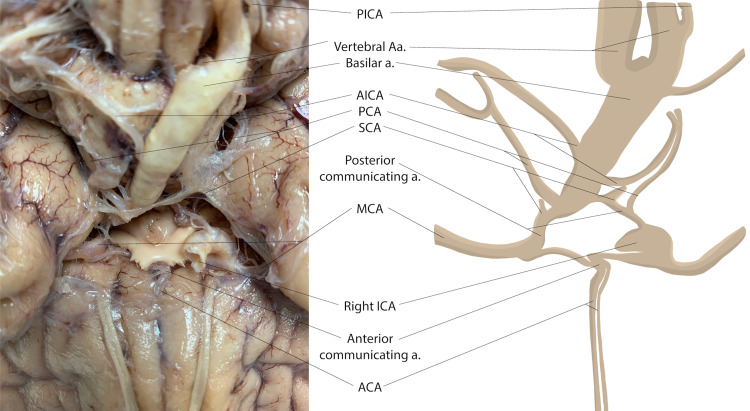
Ventral view of the brain with Circle of Willis. View of the intact Circle of Willis with absent left internal carotid artery. (Line art courtesy of Sara DeHaan.)

Upon craniotomy and removal of the brain, the vertebral and basilar arteries were notably enlarged, as was the contralateral (right) internal carotid artery. The left carotid canal was absent in the left middle cranial fossa, while the sphenoid foramen was easily identified. Aside from marked hyperplasia of the vertebral, basilar, and right internal carotid artery, the Circle of Willis and related branches were present without notable variation (Figure 2). No additional anastomoses or persistent fetal intracranial arteries were identifiable.

A literature review identified reports of agenesis of the internal carotid arteries, noting various types “A­-F” [4, 7]. Types A and B describe a broken or incomplete Circle of Willis. Type C represents bilateral underdevelopment of the internal carotid arteries, while type E is bilateral hypoplasia of the internal carotid arteries. Type D includes an anastomosis of the cavernous portion of the internal carotid arteries. Type F includes a rete mirabile network of vasculature connecting the external carotid artery with a short internal carotid artery within the cranium. Additional types reported include persistent segmental or developmental arteries that often branch from the basilar artery to form an internal carotid artery within the cranium. 

## Discussion

In this report, we have described a case of a complete aplasia of the left internal carotid artery with an intact Circle of Willis. A non-systematic search of relevant literature was performed using keywords including “internal carotid artery”, “aplasia”, and/or “agenesis” on the NIH NLM NCBI “PubMed” database and Google Scholar, with relevant articles identified by title and abstract. Furthermore, “Bergman’s Comprehensive Encyclopedia of Human Anatomic Variation” [6] was consulted. The case we present here differs from identified variants previously described in the scientific literature [4, 6-7]. The case presented includes a complete Circle of Willis, no persistent developmental branches or unusual intracranial anastomoses, or does it include an intracranial portion of the absent left internal carotid artery. This excluded association with the reported Types A, B, C, and E. Instead, the left portion of the Circle of Willis continues from the middle cerebral artery to merge directly with the posterior communicating artery posteriorly and the anterior cerebral artery anteriorly. In this case, there were no cavernous or intraosseous/petrous portions of the left internal carotid artery. This precluded the possibility of Type D and Type F variants.

Without any portion of the internal carotid artery, including the cervical, petrous, or cavernous portions, the question regarding blood supply of the orbit, eye, and hypophysis arise. Typically, the hypophysis is supplied from the superior and inferior hypophyseal arteries which branch from the cerebral and cavernous portions of the internal carotid artery. The orbit and eye are supplied through the ophthalmic artery, branching from the cerebral portion. Of note, no ophthalmic artery was observed branching from the Circle of Willis or any cerebral artery, suggesting the morphologically normal left orbit and eye were supplied from another source. We presume this source in this case to be a branch of the maxillary artery ascending through the inferior orbital fissure; however, it is reportedly more common to find a middle meningeal artery origin of the ophthalmic artery [2]. Another possibility involves a persistent trigeminal artery from development, which was not present in this case. We presume the source of blood supply to the pituitary was likely from branches of the contralateral internal carotid artery within the cavernous sinus, as anastomoses with the contralateral pituitary blood supply are common [6]. In the case of bilateral agenesis of the internal carotid artery, the blood supply from dural branches of the middle meningeal artery is feasible, as anastomoses at this point have been known to occur [6, 8].

Clinically, the absence of the internal carotid artery has been correlated with a number of conditions, likely contingent upon the precise nature of the absence and effectiveness of any collateral circulation developed. Pulsatile tinnitus, ischemic stroke, migraine, Horner’s syndrome, and subarachnoid hemorrhage have all been reported in the scientific literature [9]. Reported single cases associated with other congenital conditions have included Goldenhar syndrome [4] and Klippel-Trenaunay syndrome [10], although the statistical correlation with such syndromes is unknown. Depending upon the exact nature of the blood supply of the hypophysis, the agenesis of the complete internal carotid artery may cause the pituitary gland to become increasingly susceptible to ischemic damage. Also, it seems likely that developmental disorders of the third pharyngeal arch would likely be associated with agenesis of the internal carotid artery and structures of similar embryologic origin, although that was not the case described herein. In addition, the variation of the internal carotid artery can impact blood flow during manual therapy intervention in physical rehabilitation [11]. 

The absence of the internal carotid artery is of critical importance for surgical considerations. While some patients with this condition may present asymptomatically, the sufficiency of cerebral blood flow may become dependent upon the vertebral artery or the contralateral, intact internal carotid artery. During procedures such as endarterectomy for the removal of atherosclerotic plaque, cerebral blood flow may become severely impacted having drastic consequences. Understanding blood supply to the pituitary gland is also critical due to the relatively common occurrence of hypophyseal tumors [9] and the limited redundancy in irrigation. As the blood supply to the orbit may be complicated and potentially traverse unusual locations such as the cavernous sinus and the dura mater, or form anastomoses with the middle meningeal artery, surgeries or thrombic events may inadvertently impact such a patient’s vision or pituitary function.

As a single case, this report has clear limitations, including its lack of ability to determine prevalence. The routine nature of the dissection for medical education prohibited prolonged and detailed investigation required to identify collateral blood supply noted here in the Discussion. Comprehensive, multi-center retrospective studies of neuroangiographic images may provide detailed information regarding the prevalence of agenesis of one or both internal carotid arteries and allow for statistical association with clinical presentations and medical conditions. Collectively, the three professional anatomists involved in this case have dissected an estimated total of 500 unique cadavers, and this is the first instance in which an internal carotid artery and carotid canal were not developed.

## Conclusions

This cadaveric case report describes a seemingly unique variant of the cerebral blood flow in association with agenesis of one of the internal carotid arteries and carotid canal. We performed a scholarly literature review, and suggest that such a condition can have drastic implications for cranial surgery and ischemic events, including the pituitary gland and eye. Multi-center studies of angiographic imaging are recommended to determine prevalence and associated clinical presentation.

## References

[1] Sethi D, Gofur EM, Munakomi S (2021). Anatomy, Head and Neck, Carotid Arteries. https://www.ncbi.nlm.nih.gov/books/NBK545238.

[2] Rosen RD, Bordoni B (2021). Embryology, Aortic Arch. http://www.ncbi.nlm.nih.gov/books/nbk553173/.

[3] Rosen IW, Mills DF, Nadel HI, Kaiserman DD (1975). Angiographic demonstration of congenital absence of both internal carotid arteries. Case report. J Neurosurg.

[4] Ottaviano G, Calzolari F, Martini A (2007). Goldenhar syndrome in association with agenesia of the internal carotid artery. Int J Pediatr Otorhinolaryngol.

[5] Given CA 2nd, Huang-Hellinger F, Baker MD, Chepuri NB, Morris PP (2001). Congenital absence of the internal carotid artery: case reports and review of the collateral circulation. Am J Neuroradiol.

[6] Zink WE, Komotar RJ, Meyers PM (2007). Internal carotid aplasia/hypoplasia and intracranial saccular aneurysms: series of three new cases and systematic review of the literature. J Neuroimaging.

[7] Bergman RA, Tubbs RS, Shoja MM, Loukas M (2016). Bergman's comprehensive encyclopedia of human anatomic variation.. Bergman's comprehensive encyclopedia of human anatomic variation. Hoboken.

[8] Li S, Hooda K, Gupta N, Kumar Y (2017). Internal carotid artery agenesis: a case report and review of literature. Neuroradiol J.

[9] Cohen JE, Gomori JM, Leker RR (2010). Internal carotid artery agenesis: diagnosis, clinical spectrum, associated conditions and its importance in the era of stroke interventions. Neurol Res.

[10] Goldstein SJ, Lee C, Young AB, Guidry GJ (1984). Aplasia of the cervical internal carotid artery and malformation of the circle of Willis associated with Klippel-Trenaunay syndrome. Case report. J Neurosurg.

[11] Thomas LC, Rivett DA, Bateman G, Stanwell P, Levi CR (2013). Effect of selected manual therapy interventions for mechanical neck pain on vertebral and internal carotid arterial blood flow and cerebral inflow. Phys Ther.

